# Association between critical care occupancy and code status decisions during resource scarcity: a retrospective cohort study

**DOI:** 10.1186/s12910-025-01299-x

**Published:** 2025-11-03

**Authors:** Stijn Bex, Lorna Guinness, Christophe Gaudet-Blavignac, Jeremy H. Martin, Jérôme Stirnemann, Thomas Agoritsas, Anne Rossel, Antonio Leidi, Olivier Grosgurin, Jean-Luc Reny, Christophe A. Fehlmann, Samia Hurst-Majno, Christophe Marti

**Affiliations:** 1 Division of General Internal Medicine, Geneva University Hospitals, rue Gabrielle Perret-Gentil 4, CH-1211 Geneva 14, Geneva, Switzerland; 2 Faculty of Public Health and Policy, London School of Hygiene and Tropical Medicine, London, United Kingdom; 3 Division of Medical Information Sciences, Geneva University Hospitals, Geneva, Switzerland; 4 Department of Radiology and Medical Informatics, University of Geneva, Geneva, Switzerland; 5 Division of Internal Medicine, Centre Hospitalier de Bienne, Bern, Switzerland; 6 Departments of Health Research Methods, Evidence, and Impact, McMaster University, Hamilton, ON Canada; 7 MAGIC Evidence Ecosystem Foundation, Oslo, Norway; 8 Division of Intensive Care Medicine, Department of Acute Care Medicine, Geneva University Hospitals, Geneva, Switzerland; 9 Division of Emergency Medicine, Department of Acute Care Medicine, Geneva University Hospitals, Geneva, Switzerland; 10 Institute for Ethics, History and the Humanities, Faculty of Medicine, University of Geneva, Geneva, Switzerland

**Keywords:** COVID-19, Code status, Implicit rationing, Intensive care, Hospital occupancy, Cohort study, Switzerland

## Abstract

**Background:**

Code status determination typically relies on the expected benefits and harms of treatment intensification and patient values and preferences. Resource availability may also influence code status decisions. During the COVID-19 pandemic, the demand for critical care often exceeded the available resources. This study investigated the association between critical care occupancy and code status decisions during the COVID-19 pandemic.

**Methods:**

We conducted a retrospective cohort study of adult patients hospitalized at Geneva University Hospital for acute COVID-19-related illness during two successive pandemic waves, in spring and autumn 2020. Multivariable logistic regression was used to analyze the association between critical care occupancy at admission and code status attribution while accounting for clinical and demographic characteristics, including age, sex, ROX index (pulse oximetry/fraction of inspired oxygen/respiratory rate), comorbidities, malignancy, nationality, insurance, and socioeconomic status.

**Results:**

A total of 2,122 patients were included in the analysis. Higher critical care occupancy was associated with an increased likelihood of being assigned an intensive care unit (ICU)-ineligible code status. The odds ratios (ORs) were 1.61 (95% CI 1.11–2.32), 1.59 (1.11–2.28) and 1.71 (1.06–2.76) for critical care occupancy levels of 100–119%, 120–139% and ≥ 140%, respectively, compared with the prepandemic baseline capacity. Other factors significantly associated with the assignment of an ICU-ineligible code status included age 70–79 years (OR 8.56; 95% CI 4.12–17.77), 80–89 years (OR 32.78; 95% CI 16.16–66.50) and ≥90 years (OR 49.04; 95% CI 23.05–104.31) and a higher comorbidity index (OR 1.22; 95% CI 1.07–1.39). Conversely, complementary hospitalization insurance was associated with lower odds of being assigned an ICU-ineligible code status (OR 0.52; 95% CI 0.29–0.92).

**Conclusions:**

Our study revealed a positive association between critical care occupancy and ICU-ineligible code status, suggesting the presence of implicit triaging during periods of high resource strain. This raises several ethical concerns, including the use of non-consensual triage criteria, lack of transparency and the risk of moral distress for healthcare professionals.

**Supplementary Information:**

The online version contains supplementary material available at 10.1186/s12910-025-01299-x.

## Background

The rapid surge in health care needs during the COVID-19 pandemic placed unprecedented pressure on health systems, raising critical questions about fair resource allocation [1]. One key issue was the distribution of scarce intensive care unit (ICU) beds. At our institution, ICU admission decisions are jointly discussed by patients, their family, and the treating physician. These discussions may occur in the Emergency Department or after admission to the acute care unit. Under normal circumstances, these decisions are based on the perceived benefit or futility of intensive care admission and patient’s values and preferences. However, during times of resource constraints, priority may be given to patients with a higher expected benefit from ICU admission although the decision process remains the same. In response to these challenges, national and international professional societies issued guidelines to assist clinicians in making triage decisions during the pandemic [2–4]. The Swiss Academy of Medical Sciences published guidelines for ICU triage during periods of resource scarcity [5]. However, these three-level recommendations were never officially applied due to regional and chronological heterogeneity in terms of resource scarcity across the country and potential political implications. These guidelines aimed to balance ethical considerations with the practical need for equitable resource allocation. Nevertheless, the decision to withhold ICU admission can also occur preemptively through the assignment of a code status that explicitly excludes ICU care based on the anticipated benefits and harms of treatment intensification [6]. Even before the pandemic, a qualitative study by Close et al. suggested that some physicians incorporate resource scarcity into code status decisions [7]. This anticipated restriction of ICU access may have a significant impact on patient outcomes. For example, a previous study in our hospital reported that 82% of hospitalized COVID-19 patients who died had not been admitted to the ICU [8]. This study aims to explore the associations between critical care occupancy, patient characteristics, and code status assignment during two waves of the COVID-19 epidemic in a Swiss tertiary care hospital.

## Methods

### Study design and data sources

This study is a secondary analysis of a retrospective cohort study evaluating trends in management and outcomes during the COVID-19 pandemic at the Geneva University Hospital (HUG) [8].

Patient data were extracted from an institutional COVID-19 database [9] and supplemented with two additional data sources. First, HUG records of daily bed occupancy during the study period were obtained. Second, patient postal addresses were linked with data on the Swiss neighborhood index of socioeconomic position (Swiss-SEP), provided by the Social and Preventive Medicine Institute of the University of Bern [10].

### Participants

The study included adult patients (≥ 18 years old) admitted to HUG with COVID-19 infection during two consecutive COVID-19 epidemic waves (February to May and October to December 2020). Patients who were hospitalized for less than 24 h and those who had explicitly refused the use of their clinical data were excluded from the analysis.

### Outcome and variables

The primary outcome was the code status documented at admission, defined as the first recorded code status within 48 h of hospitalization. If no new code status was documented during this period, the last recorded code status prior to hospitalization was used, as it is generally considered valid unless modified. Four possible code statuses were identified. “Full code” means that all possible care, including cardiopulmonary resuscitation (CPR), would be provided. If no CPR was to be provided, a patient would be coded as “DNAR” (do not attempt resuscitation), which could be further specified according to whether a patient was ICU-eligible or not, although some DNAR codes lacked such specifications. For our analysis, we dichotomized the code status into ICU-eligible code (full code, DNAR ICU-eligible and DNAR without further specification) and ICU-ineligible code. Based on clinical experience in our hospital, patients with DNAR codes lacking further specification were categorized as ICU-eligible. If no code status was documented, patients were considered full code.

The primary exposure of interest was critical care capacity strain, combining intensive care unit and intermediate care unit (IMCU) occupancy. Both units admitted critically ill patients throughout the COVID-19 pandemic [11], with noninvasive respiratory support (high-flow nasal oxygen and noninvasive ventilation) primarily delivered in the IMCU and invasive orotracheal ventilation provided in the ICU [8]. Critical care occupancy was calculated via a combined critical care occupancy index (CC-OI), derived by dividing the daily number of occupied beds by a fixed denominator representing the baseline capacity on March 1, 2020, before surge capacity was initiated.

The morbidity data included 11 comorbidity categories from the Charlson Comorbidity Index [12]. Scores were categorized as 0, 1, 2, 3 or ≥ 4. The ROX score (pulse oximetry/fraction of inspired oxygen/respiratory rate) was used as a proxy of clinical respiratory severity, with lower values indicating greater severity. This score has been shown to be associated with mortality in COVID-19 patients [13].

Data on complementary health insurance, specifically private hospitalization insurance, were also included. This insurance grants patients access to private hospital admission and, in public hospitals, to dedicated private care units. These units, which do not include the ICU or IMCU, provide enhanced accommodations and access to senior physicians, who receive additional honoraria for taking care of patients with private hospitalization insurance.

Socioeconomic status was assigned geographically to participants residing in Switzerland. Home addresses were geocoded and matched to the nearest Swiss-SEP score coordinate [10].

### Statistical methods

We report descriptive patient statistics stratified by binary code status, with mean values and standard deviations for continuous variables and absolute and relative frequencies for categorical variables. Statistical significance for between-group differences was tested via the chi-square test for categorical variables and the student’s t-test for continuous variables.

Unadjusted associations were computed between all exposure variables and the dependent variable (code status).

We used multivariable logistic regression to examine whether the critical care occupancy index (CC-OI) was associated with code status after adjustment for age, sex, comorbidities, malignancy, ROX index, nationality, socioeconomic status and insurance status. Age, comorbidities and malignancy may be potential markers of futility, whereas the ROX index is a marker of disease severity among COVID-19 patients. Patients with missing code statuses were considered full code, which corresponds most closely to our clinical experience. Odds ratios were used to describe the associations of each level of CC-OI with code status, and the Wald test was used to evaluate statistical significance. Post hoc sensitivity analyses were carried out to test the robustness of the results to alternative modeling assumptions. First, two alternative ways of handling missing data were carried out: multiple imputation using chained equations for all missing data and complete records analysis. Second, we explored an alternative model assumption using a reference CC-OI of < 80% instead of < 100%, since it could be argued that this lower threshold corresponds better to normal practice. Third, we excluded patients with the outcome “DNAR without further specification” rather than considering them as ICU-eligible. Finally, we used the CC-OI as a continuous variable, and its unadjusted association with code status was graphically represented via a logistical regression model with restricted cubic splines for the exposure.

We conducted a survival analysis of patients who were admitted with an ICU-eligible code status, in which survival was defined as maintaining an ICU-eligible code status and non-survival was defined as downgrading to an ICU-ineligible code status. A period of one week following hospital admission was considered. Kaplan‒Meier curves were computed to compare the code status over time in two cohorts of patients: those admitted on days when the forward 7-day average CC-OI was < 100% or ≥ 100%. A log-rank test was used for comparison of the two curves. Stata/IC version 16.1 was used to carry out the statistical analyses.

## Results

### Participants

.

A total of 2122 patient records were retrieved and analyzed. The average age was 67.6 years, with 55.8% male participants. The majority (59.8%) of patients had at least one comorbidity. Among all hospital admissions, 70% occurred when the CC-OI was 100% or greater. The socioeconomic status of the population was slightly skewed toward a higher status than that of the overall Swiss population. The population characteristics are provided in Table 1.

**Table 1:** Patient characteristics and critical care occupancy on the day of admission

Characteristics	Category	Total (*n* = 2122) (%)	ICU-eligible code (*n* = 1435) (%)	ICU-ineligible code (*n* = 362) (%)	Code status missing (*n* = 325) (%)	*p*- value *
Sex	Male	1185 (55.8%)	854 (59.5%)	192 (53.0%)	139 (42.8%)	< 0.001
	Female	937 (44.2%)	581 (40.5%)	170 (47.0%)	186 (57.2%)	
Age (mean, SD)	N/A	67.6, SD: 18.1	67.4, SD: 16.0	83.9, SD: 8.5	50.3, SD: 10.3	< 0.001
Age category	< 60	676 (31.9%)	453 (31.6%)	10 (2.8%)	213 (65.5%)	< 0.001
	60–69	330 (15.6%)	270 (18.8%)	6 (1.7%)	54 (16.6%)	
	70–79	458 (21.6%)	348 (24.3%)	69 (19.1%)	41 (12.6%)	
	80–89	485 (22.9%)	279 (19.4%)	190 (52.5%)	16 (4.9%)	
	≥ 90	173 (8.2%)	85 (5.9%)	87 (24.0%)	1 (0.3%)	
Comorbidity index	0	853 (40.2%)	585 (40.8%)	65 (18.0%)	203 (62.5%)	< 0.001
	1	596 (28.1%)	415 (28.9%)	120 (33.2%)	61 (18.8%)	
	2	409 (19.3%)	269 (18.8%)	99 (27.4%)	41 (12.6%)	
	3	163 (7.7%)	104 (7.3%)	47 (13.0%)	12 (3.7%)	
	≥ 4	101 (4.8%)	62 (4.3%)	31 (8.6%)	8 (2.5%)	
Malignancy	No	2025 (95.4%)	1376 (95.9%)	334 (92.3%)	315 (96.9%)	0.005
	Yes	97 (4.6%)	59 (4.1%)	28 (7.7%)	10 (3.1%)	
SSEP quintile (5 = highest)	1	393 (18.5%)	274 (19.1%)	55 (15.2%)	64 (19.7%)	0.062
	2	321 (15.1%)	209 (14.6%)	54 (14.9%)	58 (17.9%)	
	3	400 (18.9%)	265 (18.5%)	68 (18.8%)	67 (20.6%)	
	4	424 (20.0%)	293 (20.4%)	73 (20.2%)	58 (17.9%)	
	5	482 (22.7%)	327 (22.8%)	99 (27.4%)	56 (17.2%)	
	missing	102 (4.8%)	67 (4.7%)	13 (3.6%)	22 (6.8%)	
Complementary insurance	No	1963 (92.5%)	1319 (91.9%)	344 (95.0%)	300 (92.3%)	0.131
	Yes	159 (7.5%)	116 (8.1%)	18 (5.0%)	25 (7.7%)	
ROX-index category (lower = more severe)	< 5	218 (10.3%)	147 (10.2%)	42 (11.6%)	29 (8.9%)	< 0.001
	5 - <10	504 (23.8%)	374 (26.1%)	94 (26.0%)	36 (11.1%)	
	10 - <15	656 (30.9%)	475 (33.1%)	112 (30.9%)	69 (21.2%)	
	15 - <20	371 (17.5%)	258 (18.0%)	62 (17.1%)	51 (15.7%)	
	≥ 20	249 (11.7%)	122 (8.5%)	46 (12.7%)	81 (24.9%)	
	missing	124 (5.8%)	59 (4.1%)	6 (1.7%)	59 (18.2%)	
Nationality	Switzerland	1291 (60.8%)	878 (61.2%)	266 (73.5%)	147 (45.2%)	< 0.001
	EU-EEA-North America	502 (23.7%)	339 (23.6%)	81 (22.4%)	82 (25.2%)	
	Other	325 (15.3%)	217 (15.1%)	13 (3.6%)	95 (29.2%)	

*SD* Standard deviation, *EU* European Union, *EEA* European Economic Area, *SSEP* Swiss neighborhood index of socioeconomic position

*difference between ICU-eligible and -ineligible code status

**Fig. 1:**
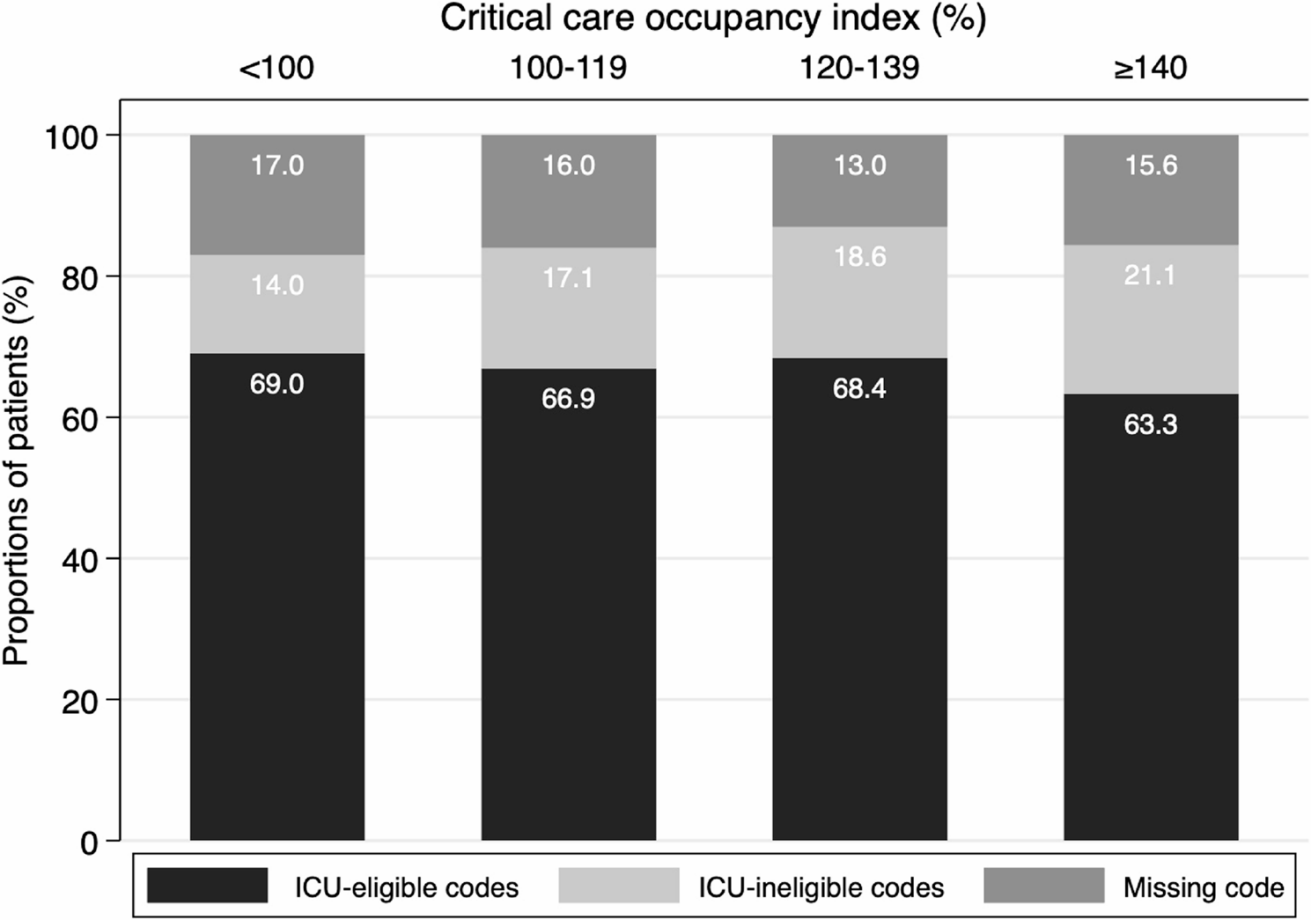
Relative frequency of code status stratified by the critical care occupancy index on the day of admission ICU: intensive care unit

Data on the relative distribution of code status at admission for the different categories of the CC-OI are provided in Fig. 1, and absolute numbers are provided in Additional file 1. Code status was missing in 15% of the records, and 22% contained at least one missing variable. Five out of 357 (1.4%) patients with an initial ICU-ineligible code status on admission were eventually admitted to the ICU, whereas 211 out of 1549 (12.0%) patients with an ICU-eligible code status were admitted.

### Bed occupancy and code status over time

**Fig. 2:**
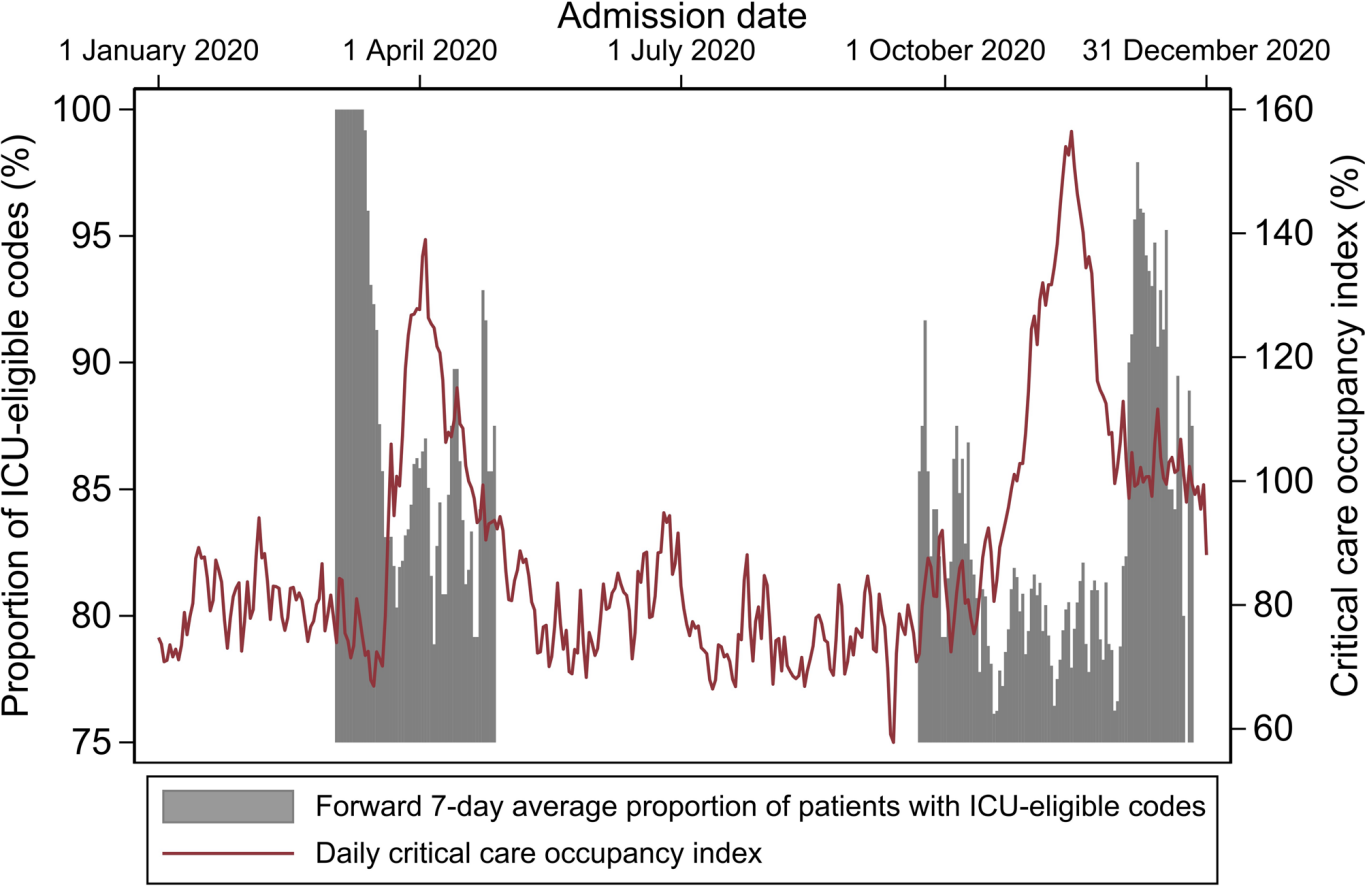
Critical care occupancy and proportion of ICU-eligible patients The forward 7-day average proportion of ICU-eligible codes displays the weighted average proportion on any given day and the following 6 days. ICU: intensive care unit

Critical care occupancy and the proportion of admitted patients with an initial ICU-eligible code status are provided in Fig. 2. The forward 7-day average proportion of ICU-eligible codes displays the weighted average proportion on any given day and the following 6 days. ICU: intensive care unit.

### Associations between critical care occupancy, patient characteristics and code status

In the unadjusted analysis, critical care occupancy, age greater than 70 years, a higher comorbidity index, malignancy and Swiss nationality were associated with increased odds of an ICU-ineligible code status. Conversely, complementary hospitalization insurance was associated with lower odds of an ICU-ineligible code status (Table 2).

**Table 2:** Association between exposures of interest and an ICU-ineligible code

Variable	Category	Unadjusted OR for ICU-ineligible code (95% CI)	*p*-value (Wald test)	Adjusted OR for ICU-ineligible code (95% CI)	*p*-value
Critical care occupancy at admission	< 100%	1			
	100–119%	1.27 (0.93 to 1.73)	0.127	1.61 (1.11 to 2.32)	0.012
	120–139%	1.41 (1.04 to 1.89)	0.025	1.59 (1.11 to 2.28)	0.011
	≥ 140%	1.64 (1.11 to 2.44)	0.014	1.71 (1.06 to 2.76)	0.027
Gender	Male	1			
	Female	1.15 (0.91 to 1.44)	0.238	1.07 (0.81 to 1.41)	0.650
Age in years	< 60	1			
	60–69	1.23 (0.44 to 3.42)	0.687	1.09 (0.38 to 3.13)	0.868
	70–79	11.81 (6.02 to 23.20)	< 0.001	8.56 (4.12 to 17.77)	< 0.001
	80–89	42.89 (22.38 to 82.21)	< 0.001	32.78 (16.16 to 66.50)	< 0.001
	≥ 90	67.37 (33.73 to 134.58)	< 0.001	49.04 (23.05 to 104.31)	< 0.001
Comorbidity index	0	1		1.22 (1.07 to 1.39) per category	0.003
	1	3.06 (2.21 to 4.22)	< 0.001		
	2	3.87 (2.76 to 5.44)	< 0.001		
	3	4.91 (3.22 to 7.50)	< 0.001		
	≥ 4	5.37 (3.28 to 8.79)	< 0.001		
Malignancy	No	1			
	Yes	2.05 (1.30 to 3.24)	0.002	1.47 (0.81 to 2.67)	0.200
SSEP quintile (5 = highest)	1	1		0.99 (0.90 to 1.09) per category	0.872
	2	1.24 (0.83 to 1.87)	0.297		
	3	1.26 (0.86 to 1.85)	0.243		
	4	1.28 (0.87 to 1.87)	0.206		
	5	1.59 (1.11 to 2.28)	0.012		
Complementary hospitalization insurance	No	1			
	Yes	0.60 (0.36 to 0.99)	0.048	0.52 (0.29 to 0.92)	0.024
ROX-index category (lower = more severe)	< 5	1		1.00 (0.89 to 1.13) per category	0.976
	5 - <10	0.96 (0.64 to 1.44)	0.846		
	10 - <15	0.86 (0.58 to 1.28)	0.462		
	15 - <20	0.84 (0.55 to 1.30)	0.433		
	≥ 20	0.95 (0.60 to 1.51)	0.827		
Nationality	Switzerland	1			
	EU-EEA-North America	0.74 (0.56 to 0.97)	0.032	0.89 (0.64 to 1.24)	0.489
	Other	0.16 (0.09 to 0.28)	< 0.001	0.58 (0.30 to 1.10)	0.097

*ICU* Intensive care unit, *OR* Odds ratio, *CI* Confidence interval, *SSEP* Swiss neighborhood index of socioeconomic position, *EU* European Union, *EEA* European Economic Area

**Fig. 3:**
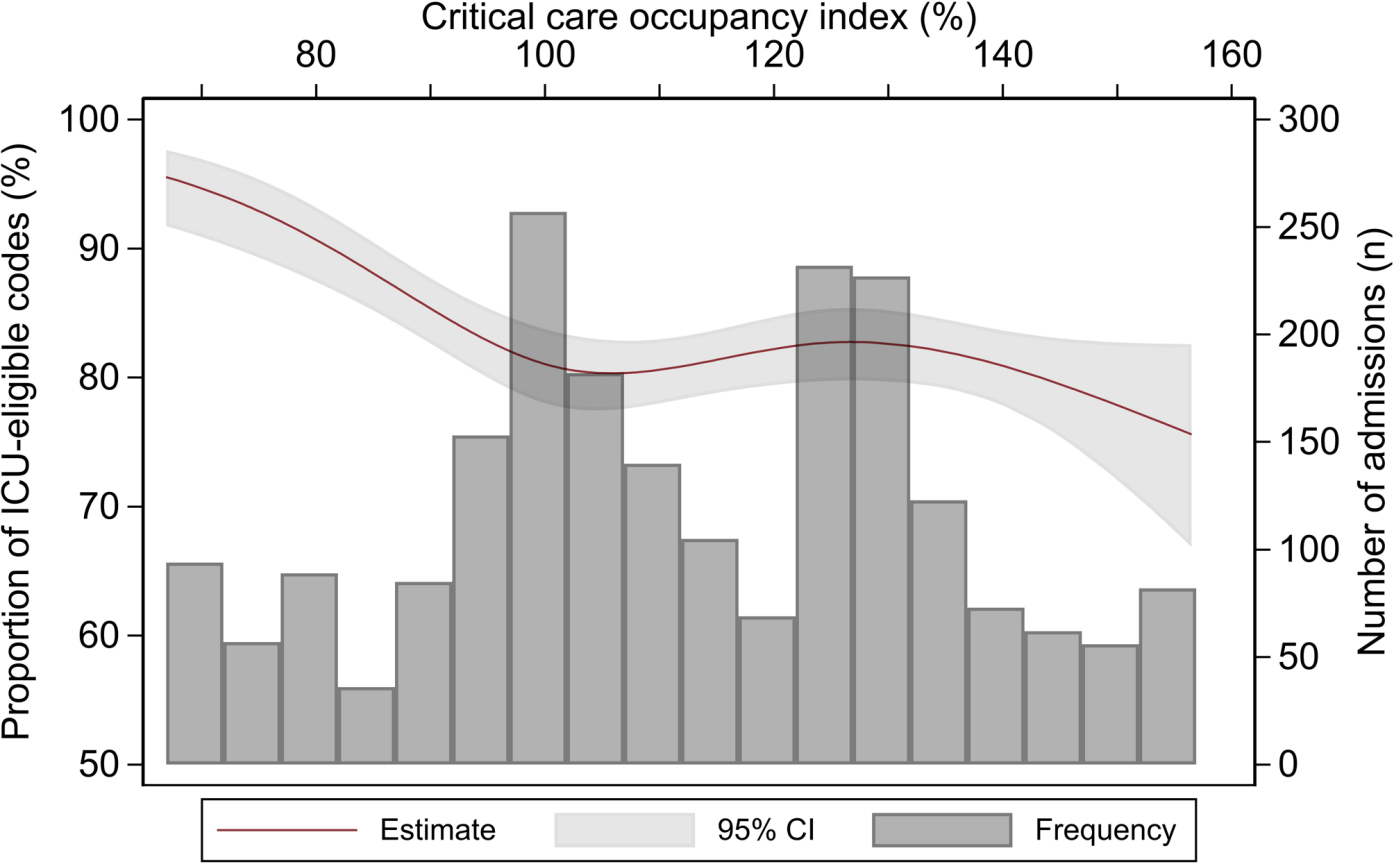
Unadjusted association of critical care occupancy with code status The shading displays the 95% confidence interval. The histogram displays the number of hospital admissions relative to the critical care occupancy index at the time of admission. ICU: intensive care unit

Figure 3 shows that the unadjusted association of CC-OI with code status displays inflection points at CC-OIs of 1 and 1.3. According to the multivariable analysis, the association between critical care occupancy and ICU-ineligible code status was significant across all occupancy intervals: the OR was 1.61 (95% CI 1.11–2.32) for the CC-OI interval of 100–119%, 1.59 (95% CI 1.11–2.28) for the 120–139% interval and 1.71 (95% CI 1.06–2.76) for the ≥ 140% interval. The associations of age, the comorbidity index and complementary insurance with code status remained statistically significant in the multivariable analysis (Table 2). Nationality and malignancy were not significantly associated with code status in the multivariable analysis. The associations of CC-OI, age, the comorbidity index and complementary insurance with code status were robust to sensitivity analyses using multiple imputation and complete records analysis for missing data (Additional files 2 and 3). The sensitivity analysis using a CC-OI of < 80% as a reference revealed an increased strength of association between CC-OI and code status (OR 4.43; 95% CI 2.06–9.54) for CC-OIs of 80–100% compared with < 80%) (see Additional file 4). The sensitivity analysis that excluded patients with the outcome “DNAR without further specification” showed slightly weaker associations between CC-OI and code status (OR 1.50; 95%CI = 0.95 to 2.35 for a critical care occupancy between 100 and 119%), (see Additional file 6).

### Kaplan‒Meier–secondary analysis

**Fig. 4:**
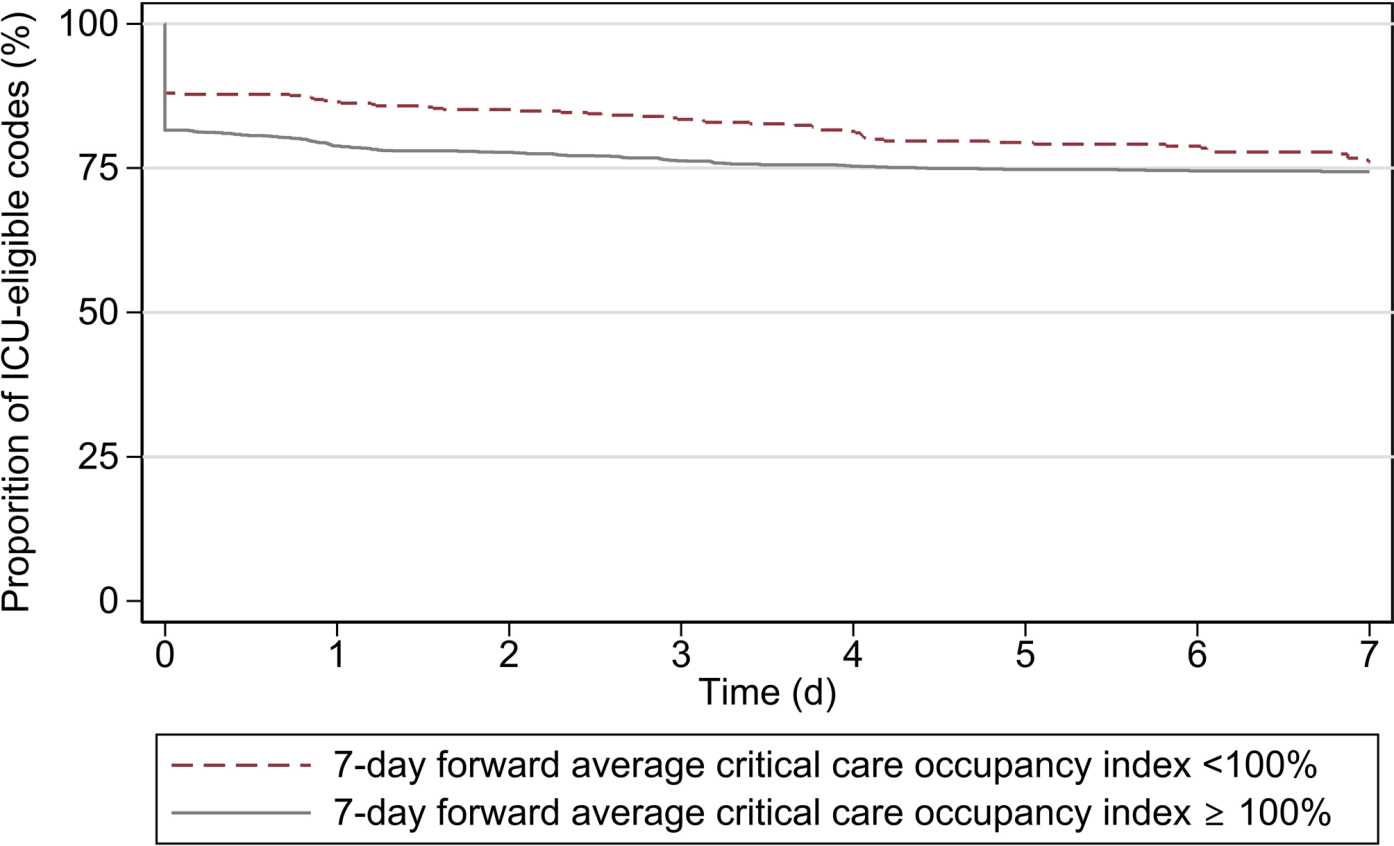
Kaplan‒Meier survival estimates of patients admitted with an ICU-eligible code Survival is defined as maintaining an ICU-eligible code within the first 7 days of hospitalization. ICU: intensive care unit

Kaplan‒Meier survival estimates comparing the retention of an ICU-eligible code status of the cohorts of patients admitted at times of < 100% and ≥ 100% forward 7-day CC-OI are presented in Fig. 4. Visually, the proportion of ICU-eligible patients decreased similarly over time in the two cohorts of patients. The log-rank test for equality of the survivor function gives a value of Chi^2^ with one degree of freedom of 0.54, corresponding to a p-value of 0.464.

## Discussion

This study shows that during the two 2020 waves of the COVID-19 epidemic at the HUG, critical care occupancy at admission was independently positively associated with ICU-ineligible code status. Age older than 70 years and a greater number of comorbidities were also associated with an ICU-ineligible code status. On the other hand, holding complementary hospitalization insurance was associated with lower odds of an ICU-ineligible code status. Survival analysis revealed no difference in the attrition rate of ICU-eligible code status during the first 7 days of hospitalization between patients admitted at < 100% CC-OIs and those admitted at ≥ 100% CC-OIs.

The findings of our study have implications for the allocation of resources in times of scarcity. A code status is supposed to be informed by patient preferences and medical utility or futility of specific interventions [14, 15]. Our findings suggest that the context-related factor of bed scarcity interferes. This finding was previously reported in a qualitative study before the COVID-19 pandemic [7]. Similarly, a retrospective Swedish cohort study recently reported a positive association between both ICU and hospital occupancy and non-admission to the ICU during the COVID-19 pandemic [16]. When code status decisions at the time of admission are influenced by bed availability or other resource constraints, this represents a form of implicit rationing of access to life-supporting care. Physicians may attempt to integrate resource constraints into decisions that are characterized by high levels of complexity and uncertainty, aiming to maximize benefits at the population level. However well-intentioned this may be, this form of bedside rationing lacks consistency and may be influenced by preexisting implicit biases [17]. The patient, or even the provider, may not be aware that resource constraints are part of the decision, which is problematic in terms of transparency and equity. Anticipated ICU bed shortages could lead to more restrictive code statuses; without any certainty that future capacity will be insufficient. This may lead to underutilization of ICU services that would be considered useful by both the patient and the health staff. Finally, the dilemmas posed by bedside rationing may lead to moral distress among healthcare providers [18]. The factors contributing to implicit rationing are complex and highly context dependent. Patients and care providers in high-income countries like Switzerland may not be familiar with situations of resource constraints. A critical step is to acknowledge implicit rationing when it occurs, which is what this study aims to contribute to. Indeed, during the COVID pandemic, the absence of official recognition of resource scarcity precluded the application of the recommendations of the Swiss Medical Science Academy regarding ICU triage during resource scarcity.

Our study did not find evidence of bed occupancy leading to faster downgrading of code status in times of high ICU/IMCU strain. This is consistent with what has previously been reported in an ICU setting [19]. This finding suggests that implicit rationing takes place at hospital admission rather than later during hospitalization.

Age was strongly associated with ineligibility for ICU admission, with a multivariable OR of approximately 8 for the 70–80 years old category and of over 30 for patients over 80 years old compared with patients under 60 years old. In a previous survey conducted at our institution, older age, comorbid conditions, patients’ values and preferences and the absence of decision-making capacity were the four major justifications provided by residents regarding DNAR orders [20]. However, the excessive weighting of chronological age has been questioned, as it might prevent older, otherwise healthy patients from benefiting from ICU admission [21]. For these reasons, frailty has emerged as an important predictor of mortality in critically ill elderly patients [22]. This could be included in ICU admission decisions together with age, comorbidities, illness severity and personal preferences, although this was not explored in our study.

Patients who held complementary hospitalization insurance had approximately half the odds of being attributed an ICU-ineligible code status (OR of 0.52). This association was present after correction for confounders, including neighborhood-based socioeconomic status and nationality. Our findings suggest a non-equitable distribution of access to ICU care, which could be the consequence of different patient or provider attitudes based on insurance status. On the patient’s side, one possible explanation is that patients with complementary hospitalization insurance are more vocal about their preferences or more prone to opt for treatment intensification. Alternatively, healthcare providers may approach code status decisions differently based on insurance status. Moreover, senior physicians might advocate more effectively for their patients to obtain scarce resources. At the population level, Danis et al. reported that uninsured patients had increased mortality and were approximately half as likely to be admitted to the ICU, but this study was not adjusted for disease severity [23]. In the North American literature, several studies previously reported an association between code status decisions and the type of health insurance [24–26]. In a multicentric retrospective study, Lyon et al. reported an association between a lack of health insurance and ICU mortality, as well as decreased use of invasive procedures such as central venous catheters, acute hemodialysis, and tracheostomy [27]. On the other hand, Epler et al. did not report any significant association between code status and insurance status among COVID-19 patients admitted to a single academic center [24].

ICU code status decision making is a complex process involving patients, relatives, physicians and other healthcare providers in charge of acute care units and critical care consultants. Unfortunately, the absence of standardized recording of the reasons for non-ICU eligible code status in our study does not allow us to determine which actors may have been influenced by insurance status.

Our study included a large sample size of 2122 patients who were consecutively included, thereby limiting selection bias. The documented associations were controlled for most of the factors previously described in the literature to be associated with code status decisions [24–26, 28–39]. Ethnicity was described as an independently associated factor in several studies, but this variable is not recorded at the HUG. The association between the main exposure and outcome was strengthened after correction for confounders, adding to the credibility of this association. Overall, 22% of our data records contained missing data, but we explored two alternative ways of handling missing data in a sensitivity analysis, which showed that the main findings were robust. Additional sensitivity analysis for alternative model assumptions revealed that the association between CC-OI and code status was even stronger when a CC-OI reference value of < 80% was used. This 80% CC-OI threshold may indeed more accurately represent the point at which bed occupancy starts to influence code status decisions, which is also suggested by the continuous analysis of the OR of CC-OI. However, we did not determine this lower threshold in advance.

Our study has several limitations. Only COVID-19 patients were included, potentially limiting the generalizability of our findings. However, the fact that all patients were hospitalized for the same diagnosis may limit potential confounding related to the type of acute illness regarding code status decisions. Additionally, only data from one hospital were studied. Since code status is likely influenced by cultural factors, the conclusions may not be generalizable to other contexts. Even though we corrected for markers of disease severity, as well as important prognostic variables such as age and comorbidities, residual confounding cannot be excluded because of the retrospective study design. For example, our recording of patient comorbidities relied on diagnostic codes in the electronic medical file, which may incompletely reflect a patient’s true comorbidity status. Some elements, such as a patient’s frailty, which is a well-established predictor of outcomes in critically ill patients, are not captured in a diagnostic code; however, they are likely to influence code status. It is worth mentioning that in our study, the bed occupancy index used a fixed number of beds as a denominator, and values > 100% did not necessarily imply the unavailability of ICU or IMCU beds.

In our main analysis, patients with DNAR orders without further specification were considered ICU-eligible, based on our clinical experience. The strength of the association between CC-OI and ICU-eligible code status was slightly reduced and statistical significance was lost after exclusion of these 302 patients, limiting the robustness of our findings. The loss of statistical significance could be explained by the 15% sample size reduction in this post-hoc sensitivity analysis.

Finally, there is no standardized recording of the reasons for attributing an ICU-ineligible code status in our institution.

## Conclusions

Our study revealed a positive association between critical care occupancy and the assignment of an ICU-ineligible code status, suggesting the presence of implicit triaging during periods of high resource strain. We also found that complementary health insurance was associated with a lower chance of an ICU-ineligible code status. This raises several ethical concerns, including the use of non-consensual triage criteria, lack of transparency, and the risk of moral distress for healthcare professionals.

## Supplementary Information

Supplementary Material 1.

Supplementary Material 2.

Supplementary Material 3.

Supplementary Material 4.

Supplementary Material 5.

Supplementary Material 6.

## Data Availability

The datasets used and/or analyzed during the current study are available from the corresponding author upon reasonable request.

## Abbreviations

CC-OI: Critical care occupancy index
COVID: Coronavirus disease
CPR: Cardiopulmonary resuscitation
DNAR: Do not attempt resuscitation
HUG: Hôpitaux Universitaires de Genève
ICU: Intensive Care Unit
IMCU: Intermediate Care Unit
OR: Odds ratio
SEP: Socioeconomic position
