# Supplementary material for: Association between critical care occupancy and code status decisions during resource scarcity: a retrospective cohort study
Source: BMC Med Ethics. 2025 Nov 3;26:156. doi: 10.1186/s12910-025-01299-x (PMC12581500; doi:10.1186/s12910-025-01299-x)
Supplement: Supplementary file 1 — Supplementary Material 1. [file 12910_2025_1299_MOESM1_ESM.docx]

**Critical care occupancy index distribution according to code status at admission.**

| Critical care occupancy index at admission | Total  (n=2122) (%) | ICU-eligible code  (n=1435) (%) | ICU-ineligible code  (n=362) (%) | Code status missing  (n= 325) (%) | p- value Chi^2^-test |
| --- | --- | --- | --- | --- | --- |
| <100% | 636 (30.0%) | 439 (30.6%) | 89 (24.6%) | 108 (33.2%) | 0.087 |
| 100-119% | 607 (28.6%) | 406 (28.3%) | 104 (28.7%) | 97 (29.9%) |  |
| 120-139% | 661 (31.2%) | 452 (31.5%) | 123 (34.0%) | 86 (26.5%) |  |
| ≥140% | 218 (10.3%) | 138 (9.6%) | 46 (12.7%) | 34 (10.5%) |  |

ICU: intensive care unit
