# Supplementary material for: Association between critical care occupancy and code status decisions during resource scarcity: a retrospective cohort study
Source: BMC Med Ethics. 2025 Nov 3;26:156. doi: 10.1186/s12910-025-01299-x (PMC12581500; doi:10.1186/s12910-025-01299-x)
Supplement: Supplementary file 2 — Supplementary Material 2. [file 12910_2025_1299_MOESM2_ESM.docx]

**Additional file 4: Sensitivity analysis using 80% as the occupancy reference.**

| Exposure | Category | Adjusted OR for non-ICU code (95% CI) | p-value adjusted OR |
| --- | --- | --- | --- |
| Critical care occupancy at admission | <80% | 1 |  |
|  | 80-99% | 4.43 (2.06 to 9.54) | <0.001 |
|  | 100-119% | 5.25 (2.46 to 11.18) | <0.001 |
|  | 120-139% | 5.22 (2.46 to 11.08) | <0.001 |
|  | ≥140% | 5.63 (2.48 to 12.74) | <0.001 |
| Gender | Male |  |  |
|  | Female | 1.07 (0.80 to 1.42) | 0.661 |
| Age category | <60 |  |  |
|  | 60 – 69 | 1.07 (0.37 to 3.07) | 0.897 |
|  | 70 – 79 | 8.41 (4.05 to 17.49) | <0.001 |
|  | 80 – 89 | 31.78 (15.64 to 64.58) | <0.001 |
|  | ≥90 | 48.14 (22.58 to 102.63) | <0.001 |
| Comorbidity index | 0, 1, 2, 3, 4, 5 | 1.22 (1.07 to 1.39) per category | 0.003 |
| Malignancy | No |  |  |
|  | Yes | 1.49 (0.82 to 2.71) | 0.188 |
| SSEP quintile (5=highest) | 1, 2, 3, 4, 5 | 0.99 (0.90 to 1.10) per category | 0.892 |
| Complementary insurance | No |  |  |
|  | Yes | 0.50 (0.28 to 0.90) | 0.002 |
| ROX-index category (lower = more severe) | <5, 5 to <10, 10 to <15, 15 to <20, ≥20 | 1.01 (0.90 to 1.14) per category | 0.814 |
| Nationality | Swiss |  |  |
|  | EU-EEA-North Am. | 0.85 (0.69 to 1.20) | 0.357 |
|  | Other | 0.55 (0.29 to 1.05) | 0.007 |

ICU: intensive care unit; OR: odds ratio; CI: confidence interval; SSEP: Swiss neighborhood index of socioeconomic position; EU: European Union; EEA: European Economic Area.
