# Supplementary material for: Association between critical care occupancy and code status decisions during resource scarcity: a retrospective cohort study
Source: BMC Med Ethics. 2025 Nov 3;26:156. doi: 10.1186/s12910-025-01299-x (PMC12581500; doi:10.1186/s12910-025-01299-x)
Supplement: Supplementary file 3 — Supplementary Material 3. [file 12910_2025_1299_MOESM3_ESM.docx]

**Additional file 3: Sensitivity analysis using complete records analysis for missing data.**

| Exposure | Category | Adjusted OR for non-ICU code (95% CI) | p-value adjusted OR |
| --- | --- | --- | --- |
| Critical care occupancy at admission | <100% |  |  |
|  | 100-119% | 1.50 (1.24 to 1.81) | <0.001 |
|  | 120-139% | 2.08 (1.74 to 2.50) | <0.001 |
|  | ≥140% | 1.72 (1.34 to 2.20) | <0.001 |
| Gender | Male |  |  |
|  | Female | 1.09 (0.94 to 1.26) | 0.268 |
| Age category | <60 |  |  |
|  | 60 – 69 | 1.04 (0.72 to 1.50) | 0.828 |
|  | 70 – 79 | 5.89 (4.56 to 7.62) | <0.001 |
|  | 80 – 89 | 18.79 (14.78 to 23.89) | <0.001 |
|  | ≥90 | 23.66 (17.64 to 31.75) | <0.001 |
| Comorbidity index | 0, 1, 2, 3, 4, 5 | 1.28 (1.20 to 1.37) per category | <0.001 |
| Malignancy | No |  |  |
|  | Yes | 1.45 (1.08 to 1.95) | 0.013 |
| SSEP quintile (5=highest) | 1, 2, 3, 4, 5 | 1.00 (0.95 to 1.05) per category | 0.958 |
| Complementary insurance | No |  |  |
|  | Yes | 0.51 (0.38 to 0.69) | <0.001 |
| ROX-index category (lower = more severe) | <5, 5 to <10, 10 to <15, 15 to <20, ≥20 | 1.17 (1.10 to 1.24) per category | <0.001 |
| Nationality | Swiss |  |  |
|  | EU-EEA-North Am. | 0.80 (0.68 to 0.95) | 0.009 |
|  | Other | 0.55 (0.42 to 0.73) | <0.001 |

ICU: intensive care unit; OR: odds ratio; CI: confidence interval; SSEP: Swiss neighborhood index of socioeconomic position; EU: European Union; EEA: European Economic Area.
