# Supplementary material for: Association between critical care occupancy and code status decisions during resource scarcity: a retrospective cohort study
Source: BMC Med Ethics. 2025 Nov 3;26:156. doi: 10.1186/s12910-025-01299-x (PMC12581500; doi:10.1186/s12910-025-01299-x)
Supplement: Supplementary file 4 — Supplementary Material 4. [file 12910_2025_1299_MOESM4_ESM.docx]

**Additional file 2: Sensitivity analysis using multiple imputation for missing data.**

| Exposure | Category | Adjusted OR for non-ICU code (95% CI) | p-value adjusted OR |
| --- | --- | --- | --- |
| Critical care occupancy at admission | <100% |  |  |
|  | 100-119% | 1.63 (1.14 to 2.34) | 0.008 |
|  | 120-139% | 1.81 (1.28 to 2.57) | 0.001 |
|  | ≥140% | 1.89 (1.19 to 3.00) | 0.007 |
| Gender | Male |  |  |
|  | Female | 1.14 (0.87 to 1.49) | 0.980 |
| Age category | <60 |  |  |
|  | 60 – 69 | 1.01 (0.40 to 2.56) | 0.980 |
|  | 70 – 79 | 6.83 (3.12 to 14.91) | <0.001 |
|  | 80 – 89 | 23.62 (11.15 to 50.01) | <0.001 |
|  | ≥90 | 34.43 (15.36 to 77.17) | <0.001 |
| Comorbidity index | 0, 1, 2, 3, 4, 5 | 1.21 (1.07 to 1.38) per category | 0.003 |
| Malignancy | No |  |  |
|  | Yes | 1.57 (0.87 to 2.84) | 0.134 |
| SSEP quintile (5=highest) | 1, 2, 3, 4, 5 | 0.98 (0.89 to 1.08) per category | 0.667 |
| Complementary insurance | No |  |  |
|  | Yes | 0.53 (0.30 to 0.92) | 0.025 |
| ROX-index category (lower = more severe) | <5, 5 to <10, 10 to <15, 15 to <20, ≥20 | 1.04 (0.92 to 1.17) per category | 0.526 |
| Nationality | Swiss |  |  |
|  | EU-EEA-North Am. | 0.85 (0.62 to 1.17) | 0.331 |
|  | Other | 0.54 (0.28 to 1.04) | 0.067 |

ICU: intensive care unit; OR: odds ratio; CI: confidence interval; SSEP: Swiss neighborhood index of socioeconomic position; EU: European Union; EEA: European Economic Area.
