# Supplementary figures and images for: Association between critical care occupancy and code status decisions during resource scarcity: a retrospective cohort study
Source: BMC Med Ethics. 2025 Nov 3;26:156. doi: 10.1186/s12910-025-01299-x (PMC12581500; doi:10.1186/s12910-025-01299-x)

**Additional file 5: Number of COVID-19 patients hospitalized over time.**


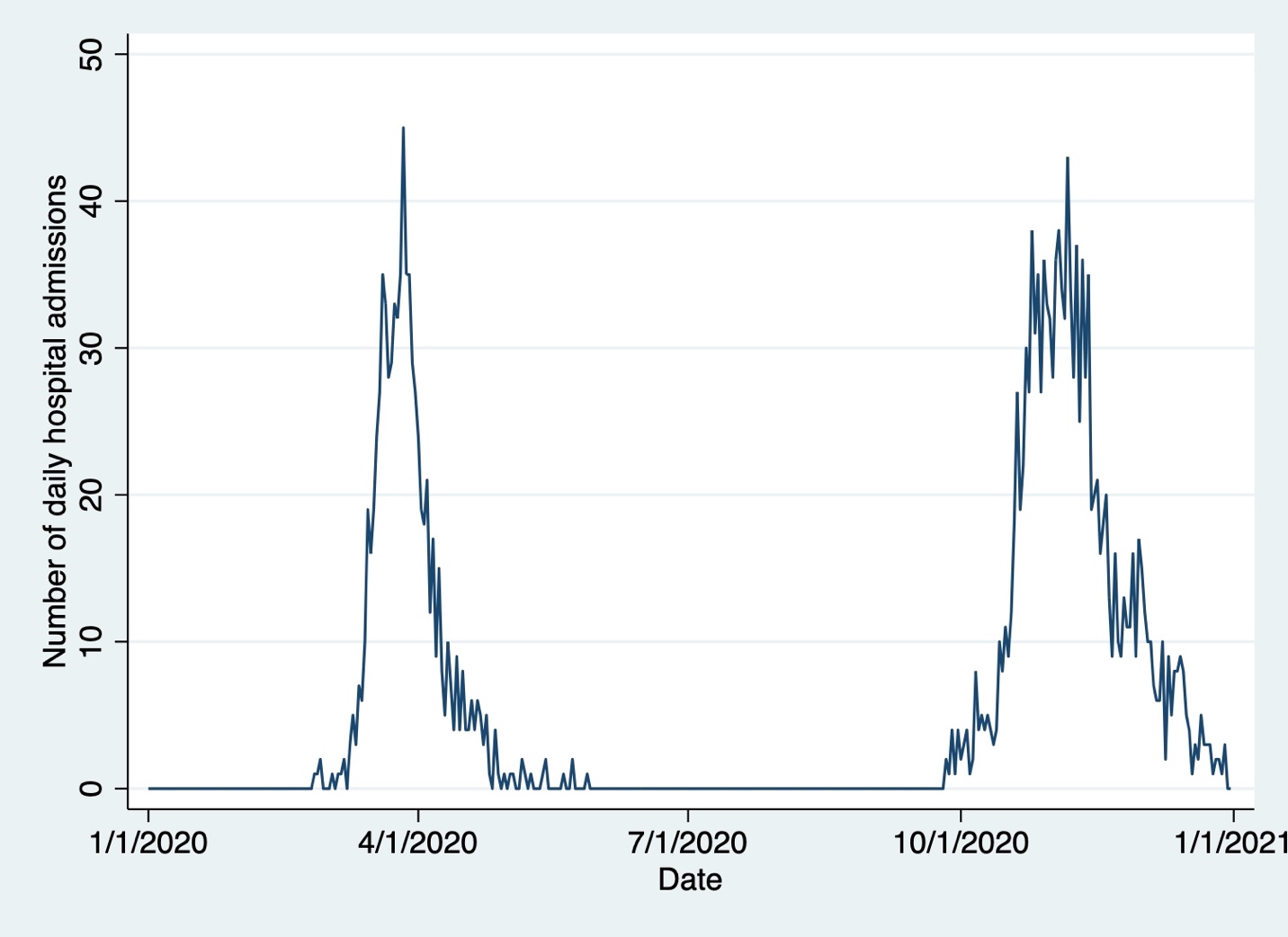

Supplement: Supplementary file 5 — Supplementary Material 5. [file 12910_2025_1299_MOESM5_ESM.docx]
