# Supplementary material for: Association between critical care occupancy and code status decisions during resource scarcity: a retrospective cohort study
Source: BMC Med Ethics. 2025 Nov 3;26:156. doi: 10.1186/s12910-025-01299-x (PMC12581500; doi:10.1186/s12910-025-01299-x)
Supplement: Supplementary file 6 — Supplementary Material 6. [file 12910_2025_1299_MOESM6_ESM.docx]

**Sensitivity analysis excluding patients with the outcome "DNAR without further specification".**

| Exposure | Category | Adjusted OR for non-ICU code (95% CI) | p-value adjusted OR |
| --- | --- | --- | --- |
| Critical care occupancy at admission | <100% |  |  |
|  | 100-119% | 1.50 (0.95 to 2.35) | 0.081 |
|  | 120-139% | 1.34 (0.87 to 2.06) | 0.185 |
|  | ≥140% | 1.42 (0.79 to 2.54) | 0.243 |
| Gender | Male |  |  |
|  | Female | 1.33 (0.95 to 1.88) | 0.101 |
| Age category | <60 |  |  |
|  | 60 – 69 | 1.05 (0.37 to 3.04) | 0.923 |
|  | 70 – 79 | 9.13 (4.36 to 19.11) | <0.001 |
|  | 80 – 89 | 59.86 (29.13 to 123.00) | <0.001 |
|  | ≥90 | 444.64 (163.03 to 1212.72) | <0.001 |
| Comorbidity index | 0, 1, 2, 3, 4, 5 | 1.37 (1.17 to 1.60) per category | <0.001 |
| Malignancy | No |  |  |
|  | Yes | 1.77 (0.86 to 3.62) | 0.119 |
| SSEP quintile (5=highest) | 1, 2, 3, 4, 5 | 1.01 (0.90 to 1.14) per category | 0.814 |
| Complementary insurance | No |  |  |
|  | Yes | 0.33 (0.17 to 0.64) | 0.001 |
| ROX-index category (lower = more severe) | <5, 5 to <10, 10 to <15, 15 to <20, ≥20 | 1.02 (0.88 to 1.18) per category | 0.808 |
| Nationality | Swiss |  |  |
|  | EU-EEA-North Am. | 0.82 (0.55 to 1.22) | 0.324 |
|  | Other | 0.47 (0.23 to 0.95) | 0.036 |

ICU: intensive care unit; OR: odds ratio; CI: confidence interval; SSEP: Swiss neighborhood index of socioeconomic position; EU: European Union; EEA: European Economic Area.
